# Comparative study of chitosan-based liquid dressing and recombinant human epidermal growth factor on acute limb skin wound healing: A randomized controlled trial

**DOI:** 10.1016/j.jpra.2025.03.018

**Published:** 2025-03-26

**Authors:** Dunyong Tan, Jiawei Guo, Xiaoqiang Chen, Jianquan Liu, Siyao Yang, Daping Wang, Wencui Li

**Affiliations:** 1 Department of Hand and Foot Surgery, Shenzhen Second People’ s Hospital (The First Hospital Affiliated to Shenzhen University), Shenzhen, Guangdong, 518028, China; 2 Guangdong Key Laboratory for Biomedical Measurements and Ultrasound Imaging, National-Regional Key Technology Engineering Laboratory for Medical Ultrasound, School of Biomedical Engineering, Shenzhen University Medical School, Shenzhen 518060, China; 3 The Medical Record Department, Shenzhen Second People’ s Hospital, Shenzhen, Guangdong, 518028, China; 4 Department of Biomedical Engineering, Southern University of Science and Technology, Shenzhen, 518055, China

**Keywords:** Wound Healing, Chitosan, EGF, Scar

## Abstract

**Background:**

Several traditional dressings may have limitation in treating wounds. A novel chitosan-based dressing designed for improved hemostasis, moisture, and sealing shows promise in wound healing. However, its efficacy and safety are yet to be sufficiently verified in patients.

**Methods:**

This randomized controlled trial enrolled 40 patients suffering from acute skin wounds in the limbs from 12/2022 to 12/2023. They were randomly divided into two groups (20 vs. 20) and received regular treatments in the Shenzhen Second People's Hospital. The experimental group was treated with chitosan-based liquid dressing, whereas the control group was treated with traditional dressing with recombinant human epidermal growth factor (rhEGF). The therapeutic effects (scar area and pigment deposition), adverse events, visual analogue scale (VAS), healing time, cost, and the patient and observer scar assessment scale (POSAS) were evaluated on days 0, 7, 14, and 28.

**Results:**

No adverse events were observed throughout the trial. On day 28, effective rate between groups were not statistically significant between the groups (70% vs. 85%, *p* = 0.256). Other parameters that were not significant included VAS (5.10 ± 1.62 vs. 6.35 ± 2.39, *p* = 0.06), healing time (8.45 ± 4.26 vs. 8.60 ± 5.44 days, *p* = 0.923), and cost (49.00 ± 22.48 vs. 57.40 ± 27.59, *p* = 0.298). However, on day 28, the patient- and observer-reported SAS of the chitosan (CS) group was significantly lower than that of the rhEGF group (12.00 vs. 9.50, z = 2.477, *p* = 0.013; 18.50 vs. 12.50, z = 2.209, *p* = 0.026; respectively), and the total POSAS (30.50 vs. 22.00, z = 2.374, *p* = 0.017).

**Conclusion:**

Compared to rhEGF, the CS-based liquid dressing showed reliable safety and equivalent performance in treating acute limb skin wounds, as revealed by improvements in healing time and rate, pain relief, and costs. Moreover, liquid dressing significantly reduced scar formation, indicating its potential in wound therapy.

## Introduction

Skin wounds are categorized into acute and chronic according to the closure time. Chronic wounds usually require more than 12 weeks to heal. Problems associated with chronic wounds include long-term treatment, therapeutic difficulties, high costs, and fair disability rates.^1^ This creates a vicious cycle that hinders wound healing and poses challenges to clinical physicians. In addition, a prolonged course of the disease will affect the quality of life of patients and increase their treatment charges, causing tremendous social and economic burdens. The market value of wound care products was estimated to be 12 billion dollars in 2020, which is predicted to reach 18.7 billion dollars by 2027.^2^

Therefore, most acute wounds require dressings that resist the invasion of external factors and promote wound healing to prevent them from becoming chronic ones. However, traditional dressings such as medical absorbent cotton gauze and Vaseline gauze feature normal hemostatic and moisturizing effects, excessive adhesion and secondary damage, and susceptibility to exogenous infection after penetration.^3^ Besides, recombinant human epidermal growth factor (rhEGF) has been widely used in wounds in clinical practice for more than two decades.^4^ It remarkably promotes cell growth, proliferation, and differentiation and has been developed into various forms.^5^ However, its efficacy is limited by its short biological half-life, physical instability, and enzyme-mediated degradability.^6^ Furthermore, the ideal wound dressing should provide moisture, remove exudation, allow ventilation, and prevent infection. Moderate adhesion, convenient replacement, and acceptable price should also be considered.^7^

Chitosan (CS) has recently gained attention in dressing development due to its biosafety, biocompatibility, biodegradability, antibacterial activity, wound healing acceleration, and significant plasticity.^8^^,^^9^ It was initially developed as a hemostatic material and related products have been approved by the US FDA for battlefield trauma hemostasis as early as 2002.^10^ To date, several in-depth studies have confirmed that CS functions throughout wound healing (hemostasis, inflammation, proliferation, and repair) and could be easily processed to cater to different wounds.^11^^,^^12^ Therefore, CS dressings appear to overcome the shortcomings of rhEGF, which are yet to be verified by relevant clinical comparative studies. Thus, we intend to compare the efficacy, safety, and cost among acute limb skin wounds by using commercial CS-based liquid and rhEGF, exploring whether CS can be a substitutional or combined option of rhEGF for acute wound treatment.

## Materials and methods

### Ethical declaration

This study is a randomized, single-blind, controlled clinical trial approved by the Medical Ethics Committee of Shenzhen Second People's Hospital (document number: 20220909001-SF01).

### Materials

For the experimental group, we applied a commercial product of chitosan quaternary ammonium biocolloid (Hebei Chuangyue Biotechnology Co., Ltd., Jixie No. 20192140001). This CS-based liquid dressing is colorless and transparent and can be sprayed directly onto superficial wounds after debridement and covered with dry, sterile gauze.

We chose another commercial product as the control group, whose main component was rhEGF (Shanghai Haohai Biotechnology Co., Ltd., National Medicine Standard No. S20010094). It is a powder that is dissolved in normal saline to create a solution with a concentration of 5000 IU/ml. This mixture was soaked with double-layer dry gauze (1 ml to 10 cm²) wrapped on debrided wounds.

Additionally, the product information on their packaging was erased to ensure the patients were treated in a single-blind manner.

### Trial design (Figure 1)

This clinical trial recruited patients with acute skin defects who visited the Department of Hand and Foot Surgery of Shenzhen Second People’ s Hospital from December 2022 to December 2023. Then, they were evaluated based on the inclusive and exclusive criteria for the following enrollment.

**Figure 1:**
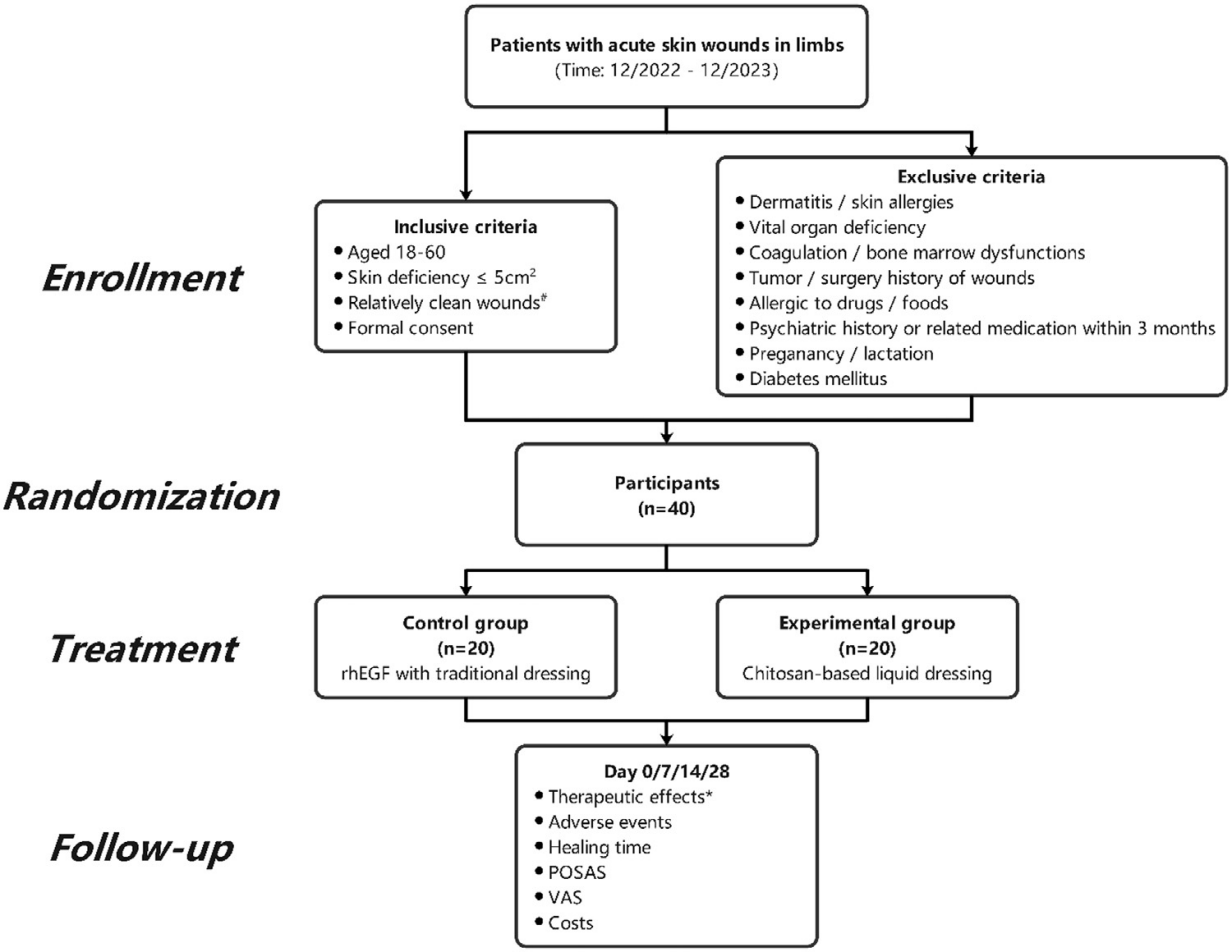
Research flowcharts #, Relatively clean wounds: No or only minimal infection of the wound (no fever or transient low-grade fever, leukocyte ≤ 12 × 10^9^/L, and no restricted-grade antibiotics were used); *, therapeutic effects:Effective rate = (excellent + good) case / total case × 100%. rhEGF, recombinant human epidermal growth factor; POSAS, patient and observer scar assessment scale; VAS, visual analogue scale.

Inclusive criteria: (i) 18-60 years old; (ii) skin defect <5 cm^2^ (not exceeding the dermis); (iii) no infection or slight infection (no fever or transient low-grade fever, leukocyte ≤ 12 × 10^9^/L, and no restricted-grade antibiotics were used); and (iv) informed consent from the patient.

Exclusion criteria: (i) History of skin diseases and drug allergies; (ii) dysfunction of essential organs (heart, liver, kidney, lung, and brain) or abnormal coagulation or bone marrow function; (iii) history of tumor and surgery in the defect area; (iv) history of allergies to drugs and foods; (v) history of psychiatric illness and use of antipsychotic medications three months ago; (vi) pregnant women and breastfeeding; and (vii) history of diabetes mellitus.

Prior to the trial initiation, detailed procedures were introduced to the patients, including the advantages and disadvantages of the two treatment methods. Patients were informed about the principle of random allocation and provided with written informed consent.

The enrolled patients were given a randomly generated number. Those with odd numbers were assigned to the experimental group and were treated with CS-based liquid dressing, while the others were assigned to the control group and treated with rhEGF. Thus, 40 cases were equally divided into two groups.

### Baseline evaluation

Baseline data were collected from all patients before treatment, including chief complaints, current medical history, past history, growth and development history, family history, and physical examination data. Additional examinations included blood routine tests, liver and kidney function tests, coagulation function tests, and fasting blood sugar levels. Then, those patients were treated by the same physician team. Standard debridement was performed to remove devitalized tissue.

### Treatment

rhEGF Group: The rhEGF freeze-dried powder was dissolved in saline to prepare a 5000 IU/ml solution. Approximately 1 ml of this solution was evenly sprayed from a distance of 10 cm from the wound. After 30 s, a dry sterile dressing (gauze) was cut to cover it, extending 0.5 cm beyond its outer edge. The dressing was changed every 3 days.

CS Group: Approximately 1 ml of the test liquid was evenly sprayed from a distance of 10 cm from the wound. After 30 s, a dry sterile dressing (gauze) was cut to fit the wound size, extending 0.5 cm beyond its outer edge. The dressing was also changed every 3 days.

When changing, the wound was sterilized with 0.5% iodine. Saline was used to moisten the dressing before removal if necessary. Dressing changes and wound assessments were performed by clinicians from the same therapeutic team. The use and replacement procedures of dressings were carried out following the instructions for each product.

### Outcome evaluation

Continuous follow-up and data collection were performed on days 7, 14, and 28 throughout the four-week trial. The data collected included: (i) Adverse reactions, (ii) total effective rate, (iii) wound healing time, (iv) patient and observer scar assessment scale (POSAS), (v) visual analogue scale (VAS) for pain, and (vi) average treatment costs.

#### Therapeutic effects (Effective rate)

The clinical efficacy and efficacy standards of the two groups were compared based on the criteria recommended by Lin et al.^13^

The efficacy standards were described as follows:(i) Excellent: The wound healed completely, skin had no noticeable tension, and color closely resembled that of healthy skin. (ii) Good: The wound healing rate was >70%, with no noticeable tension and a natural appearance. (iii) Poor: The wound healing rate was ≤70%, or there was evident tension and scarring on the skin. The effective rate was defined as the ratio of excellent and good cases with respect to total cases.^13^

#### Scar appearance

We assessed the appearance and area of scar hyperplasia after wound healing. The wound and scar areas were measured using the ImageJ software. Their ratio was measured and calculated according to the scale bar in the photographs.

#### Wound healing rate and time

We recorded the time for complete re-epithelialization of the wound. Re-epithelialization refers to resurfacing a wound with new epithelium.^14^ The healing rates and average healing time of the two groups at different time points were compared. This assessment was based on the visual observation by an associate chief physician who had not participated in this trial previously.

#### Scar assessment scale (POSAS)

The POSAS includes observer and patient scales.^15^

(i) Observer Scale: Scoring items include vascularity, color, thickness, surface roughness, flexibility, and surface area. Each item is scored from 1 (similar to normal skin) to 10 (the worst scar imaginable).
(ii) Patient Scale: Scoring items include pain level, itching level, color, thickness, softness, and self-perception. Scoring principles are aforementioned.

Additionally, both scales include an overall opinion score of 1 to 10. All parameters are compared to those of the normal skin in the contralateral limb.^15^ The total POSAS comprises observer- and patient-reported scores. Higher scores indicate more severe scarring. These scores were then calculated and compared between the two groups.

#### Visual analogue scale for pain

Patients rated their pain on a VAS from 0 to 10 based on their perceived pain level. A higher score indicates more severe pain.^16^ The VAS scores for both groups were recorded at each time point (0, 7, 14, and 28 days). The average VAS scores for the two groups throughout the treatment process were then calculated and compared.

#### Average treatment cost

The total treatment costs incurred by the two groups of patients throughout the entire treatment process were recorded and compared.

#### Adverse events

Both groups of patients were monitored for signs of infection, local skin redness, itching, swelling, papules, blisters, or other systemic severe adverse reactions during the treatment. Any adverse events in the subjects during the trial were recorded and analyzed.

### Statistical analysis

The statistics were collected and analyzed in a patient-blind manner. The statistical analyst needed to be informed of treatment details. The variable annotations of the treatment regimens were obtained only after statistical analysis. An independent physician not involved in this trial completed the follow-up surveys and result evaluations.

Statistical analysis was performed using the SPSS 25.0 software. Quantitative data were expressed as mean ± standard deviation (SD) or median (Q1 and Q3). Normality and variance homogeneity tests were conducted on the quantitative data. If the data met normality and variance homogeneity assumptions, comparisons were made using the Student's *t*-test. If these assumptions were not met, the Mann–Whitney U test was used. Count data were analyzed using the Pearson's chi-squared test and expressed as the number of cases (%). Statistical significance was recognized when *p* < 0.05.

## Results

### Baseline data of the participants

Overall, 40 patients with acute skin defects were included in this study and randomly distributed to the rhEGF and CS groups, respectively. Their medical history, physical examination, and laboratory tests met the inclusion and exclusion criteria. There were no significant differences in gender proportion, age, height, and body mass between the groups. Before the intervention, no existing infection, local skin redness, itching, swelling, papules, and blisters were observed (Table 1).

**Table 1:** Baseline data of the participants

	rhEGF (n = 20)	CS (n = 20)	Statistics	*p*
Gender (Male/female)	14/6	15/5	0.125^a^	0.723
Age (y)	33.60 ± 13.46	34.60 ± 11.73	0.251^b^	0.804
Height (cm)	169.40 ± 8.36	170.05 ± 5.87	0.284^b^	0.778
Body mass (kg)	64.20 ± 9.31	65.90 ± 9.33	0.577^b^	0.567
BMI (kg/m^2^)	22.32 ± 2.43	22.76 ± 2.70	0.538^b^	0.594

Data are represented as mean ± SD. a: Pearson's chi-squared test, χ^2^ value. b: non-paired independent Student's *t*-test, t value.

### Effective rates, healing rates, and healing time between two groups showed no significant difference

**Figure 2:**
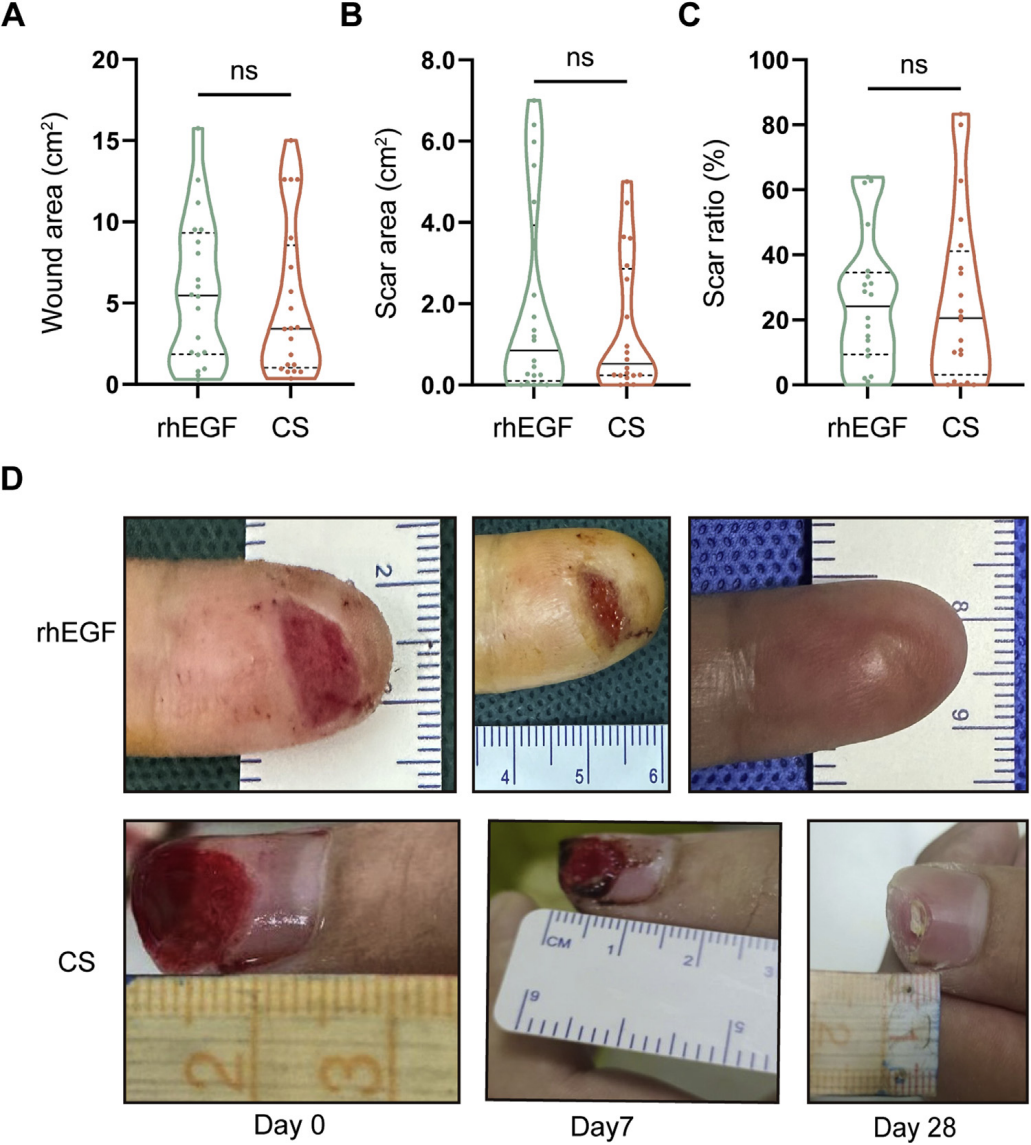
Intergroup comparison of healing assessment The wound area (A) and scar area (B) between the rhEGF and CS groups at the endpoint. (C) The representative images of the rhEGF and CS groups at different time points. Data are represented as median with Q1 and Q3, ns: not significant, non-paired independent Student's *t*-test.

The initial skin defects, final scar area, and percentage of scars between the patients treated using rhEGF and CS showed no significant difference (5.45 vs. 3.43 cm^2^, z = 0.486, *p* = 0.629; 0.85 vs. 0.53 cm^2^, z = 0.793, *p* = 0.433; 0.205 vs. 0.242, z = 0.352, p = 0.725, respectively), neither the effective rate based on the ratio of them (70% vs. 85%, *p* = 0.256) (Figure 2A, 2B, and 2C, Table 2). Besides, the healing rates at different time points (day 7/14/28) showed no significant differences, neither did the average healing time (8.45 ± 4.26 vs. 8.60 ± 5.44 days, *p* = 0.44) (Table 3). The healing wounds also displayed similar appearances among the patients who underwent two therapies (Figure 2C).

**Table 2:** Intergroup comparison of the therapeutic effects

	rhEGF (n = 20)	CS (n = 20)	Statistics	*p*
Excellent rate	70% (14/20)	85% (17/20)	1.290^a^	0.256
Excellent	45% (9/20)	45% (9/20)	/	/
Good	25% (5/20)	40% (8/20)	0.456^a^	0.500

a: Pearson's chi-squared test, χ^2^ value.

**Table 3:** Intergroup comparison of the healing rate and time

	rhEGF (n=20)	CS (n=20)	Statistics	*p*
Healing rate (%)				
7 d	60% (12/20)	70% (14/20)	0.440^a^	0.741
14 d	90% (18/20)	90% (18/20)	/	/
28 d	100% (20/20)	100% (20/20)	/	/
Healing time (d)	8.45 ± 4.26	8.60 ± 5.44	0.097^b^	0.923

Data are represented as mean ± SD. a: Pearson's chi-squared test, χ^2^ value. b: non-paired independent Student's *t*-test, t value.

### Treatment of CS-based liquid dressing reduced scar formation than traditional dressing with rhEGF

**Figure 3:**
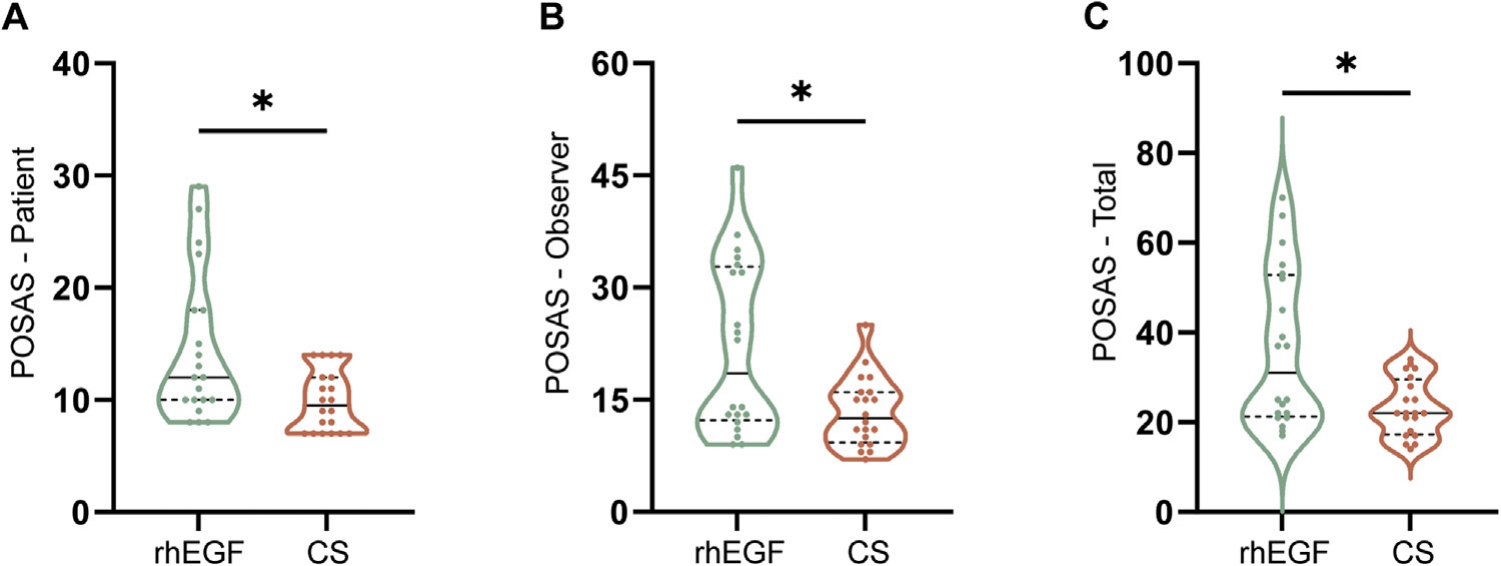
Intergroup comparison of the patient and observer scar assessment scale The patient-reported (A), observer-reported (B), and total (C) POSAS. Data are represented as median with Q1 and Q3, *: *p* < 0.05, Mann–Whitney U test.

Scar tissue is a negative factor in quality of life and can remain even after wound healing. We next evaluated the scar situation at the endpoint using POSAS. The results demonstrated that POSAS was significantly lower in the CS group, as reported by patients or observers, respectively (12.00 vs. 9.50, z = 2.477, *p* = 0.013; 18.50 vs. 12.50, z = 2.209, *p* = 0.026) (Figure 3A and 3B). Moreover, the total POSAS showed a consistent tendency (30.50 vs. 22.00, z = 2.374, *p* = 0.017) (Figure 3C).

### CS-based liquid dressing was equivalent to traditional dressing with rhEGF in pain relief and financial cost

**Figure 4:**
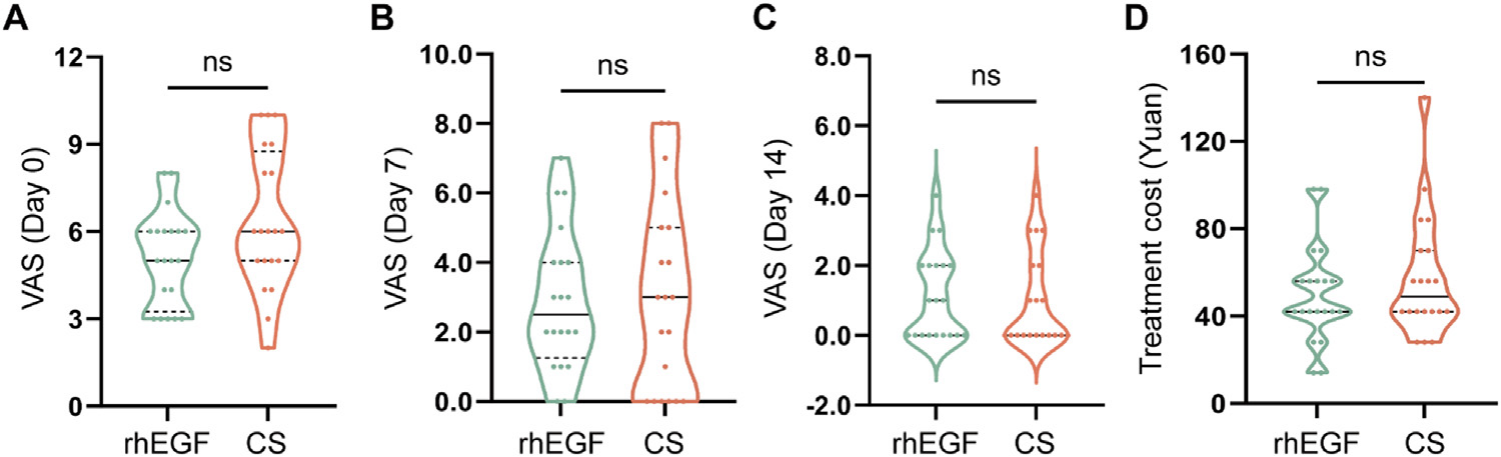
Intergroup comparison of pain and cost (A-C) The VAS of the patients on days 0, 7, and 14. (D) The total cost throughout the treatment. Data are represented as median with Q1 and Q3, ns: not significant, non-paired independent Student's *t*-test.

To compare the efficacy of pain relief between the two groups, we assessed the pain degrees of the patients at different time points. The VAS results did not show any statistical difference on days 0, 7, and 14 (6.35 ± 2.39 vs. 5.10 ± 1.62, *p* = 0.06; 3.05 ± 2.76 vs. 2.90 ± 2.00, *p* = 0.845; and 1.00 ± 1.34 vs. 1.15 ±1.268, *p* = 0.718, respectively) (Figure 4A-4C). For the financial evaluation, the median treatment cost of CS-based liquid dressing was slightly higher, with no statistical significance (49.00 ± 22.48 vs. 57.40 ± 27.59, *p* = 0.298) (Figure 4D). Additionally, no adverse events were observed during the treatment or follow-up phases (data not shown).

## Discussion

Skin is the largest organ that protects the body from external pathogens and is vulnerable to infections during acute defects. A complex repair response will be initiated after injuries, compromising several orchestrated phases, including hemostasis, inflammation, angiogenesis, re-epithelialization, and remodeling.^17^^,^^18^ Growth factors such as platelet-derived growth factor and EGF are highly involved in this process.^18^ Thus, rhEGF has been developed to promote wound healing with limited efficacy due to its intrinsic features as a bioactive agent. Recently, CS and its derivatives have emerged in wound treatment and demonstrated attractive effects.^19^, ^20^, ^21^

To date, no research has been conducted to compare the difference between CS-based liquid dressing and rhEGF. Our study applied a randomized single-blind control protocol to provide this comparison by evaluating the effective rate, healing time, VAS, SAS, cost, and adverse events. First, CS-based liquid dressing implied safety in clinical practices regardless of the limited trial size. We did not receive feedback on discomfort throughout the trial, confirming its acceptance in the treatment. However, no significant difference was observed in the healing rate or time. It is reasonable because rhEGF directly affects the regeneration process, such as angiogenesis, chemotaxis, and granulation formation.^18^ As a biological, the biosafety of protein products should be considered. Nevertheless, the CS ingredient reduces this risk with a similar outcome, which is more inspiring. Besides, the rhEGF is characterized by short-term sustenance, uncontrolled release, and enzyme-mediated degradation, driving the requirement of a combinative therapy.^22^ Thus, various biomaterials have been developed to establish a more precise and effective system. Recently, rhEGF has been integrated into the carrier system to exert robust healing effects. For example, Wang et al. designed a porous hydrogel-loaded rhEGF to restrain infection and promote skin wound healing.^23^ Nanofibrous dressings and microneedles equipped with rhEGF significantly shorten the time to wound closure and re-epithelialization in a mice model.^24^^,^^25^ Similar performance of rhEGF has been verified in diabetic foot and oral damage, indicating its wider application.^26^^,^^27^

Concurrently, therapeutic budgets expand and cause heavier financial burden. However, our results showed that CS-based liquid dressing did not result in an apparent spending increase, guaranteeing that it could replace or be combine with rhEGF without imposing excessive financial constraints.

Notably, the POSAS of the patients treated with CS-based liquid dressing was significantly lower (z = 2.374, p = 0.017). For the mechanism, CS may decompose excess collagen and reduce fibroblast and myofibroblast generation.^19^^,^^28^ However, CS could form a gel layer on the wound surface within 1-2 min, helping to stop bleeding and prevent bacterial invasion. In addition, CS, rather than gauze, could feasibly drain the exudation around the wounds, preventing unnecessary moisture that aids in pathogenesis and expansion. It additionally allowed physicians to evaluate wound healing more comfortably than with traditional dressing, which may cause painful dressing exchange due to tight adhesion.

In terms of pain relief, the two interventions showed similar effects, supported by the insignificant differences in VAS score. However, we speculated that this result could be attributed to different mechanisms. In the rhEGF group, the promotional effects of local granulation might alleviate acute hyperalgesia, leading to VAS reduction.^29^ In the CS group, rapid barrier formation might ensure the blockage of external stimuli, thereby functioning as an analgesic.^30^ In addition, its anti-inflammatory effects may also help relieve pain.^31^

The study limitations included the following aspects. First, complete double-blind observation could be hardly achieved because of the explanation of the different interventions. Second, the relatively small sample size generated inevitable bias existence. Third, more objective parameters should be adopted to ensure more comprehensive conclusions.

## Conclusion

For treating acute limb skin defects, CS-based liquid dressing is equivalent to rhEGF in wound healing efficacy and pain relief performance with similar costs. The CS-based liquid dressing is better for scar prevention, indicating its substitutional or combinative potential with rhEGF therapy.

## Competing interests

The study was sponsored by Hebei Chuangyue Biotechnology Co., Ltd, who did not participate in this study directly. None of the listed authors were affiliated with this institution. All remaining authors have declared no conflicts of interest.
